# 645. Rapid Diagnosis of Disseminated *Mycobacterium kansasii* infection in Renal Transplant Recipients Using Plasma Microbial Cell Free DNA Next Generation Sequencing

**DOI:** 10.1093/ofid/ofab466.842

**Published:** 2021-12-04

**Authors:** Tosin Ogunsiakan, Kristen D Fajgenbaum, Gautam Phadke, Thomas Montgomery, Kiran Gajurel

**Affiliations:** 1 Carolinas Medical Center, Atrium Health, Charlotte, NC; 2 Atrium Health - Carolinas Medical Center, Charlotte, NC; 3 Metrolina Nephrology, Charlotte, NC; 4 Atrium Health, Charlotte, NC; 5 Carolinas Medical Center, Charlotte, NC

## Abstract

**Background:**

Disseminated *Mycobacterium kansasii* infection is rare in kidney transplant recipients. The diagnosis may not be suspected readily due to non-specific clinical presentation. The diagnosis and treatment can be further delayed due to poor sensitivity of culture (especially of extra-pulmonary sites) and slow growth in culture media. Accurate and rapid diagnosis of disseminated *M. kansasii* infections in transplant recipients is important for antimicrobial management.

**Methods:**

Two cases of disseminated *M. kansasii* infections with unusual presentation in which rapid diagnosis was made using the Karius test (KT) are presented. The KT is a CLIA certified/CAP-accredited next-generation sequencing (NGS) plasma test that detects microbial cell-free DNA (mcfDNA). After mcfDNA is extracted and NGS performed, human reads are removed, and remaining sequences are aligned to a curated database of >1400 organisms. Organisms present above a statistical threshold are reported.

**Results:**

Case 1: A 31-year female kidney transplant recipient presented with a thyroglossal duct cyst, as well as swelling of her right metacarpophalangeal joint and left 3rd finger. AFB culture of the thyroglossal cyst aspiration done on post admission day (PAD) 2 took 27 days to be identified as *M. kansasii* (on PAD 29) whereas plasma sent for KT on PAD 5 reported a positive test for *M. kansasii* at 284 molecules/microliter (MPM) in 4 days (on PAD 9). Case 2: A 59-year male kidney transplant recipient presented with generalized weakness, arthralgia, pericardial effusion, cytopenia, weight loss and intermittent fevers. Plasma sent for KT on PAD 12 was reported positive for *M. kansasii* at 1314 MPM in 3 days (on PAD 15). PET CT done simultaneously was consistent with an infection of an old AV graft in the left upper extremity. The AFB culture of the resected graft was confirmed as *M. kansasii* in 22 days on PAD 36. After the KT was available (before confirmation of *M. kansasii* on culture), the first patient underwent modification of empiric treatment and the second patient was started on specific treatment for *M. kansasii.*

Table of *M. kansasii* cases

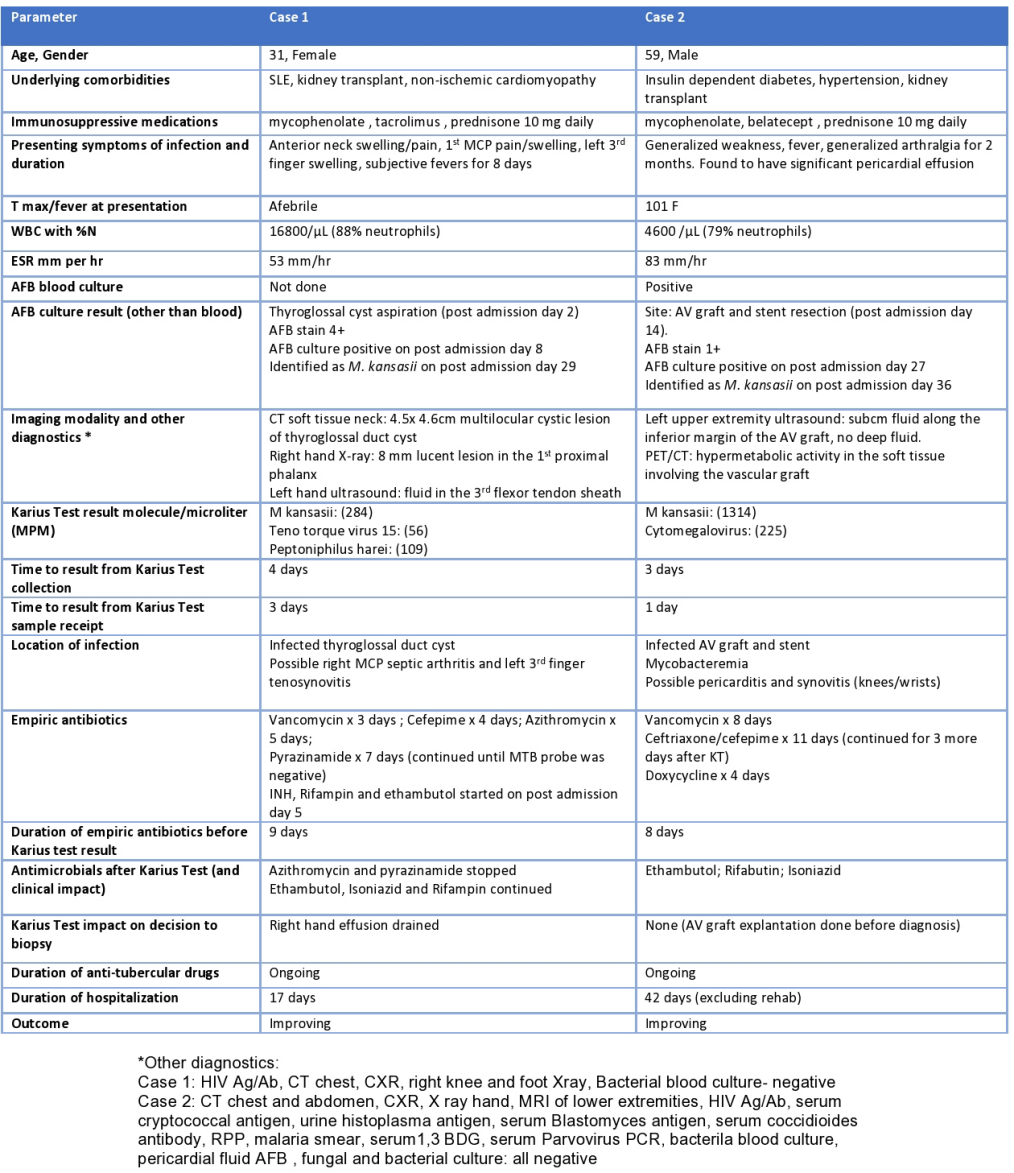

Rapid diagnosis of disseminated *M. kansasii* infection

**Conclusion:**

Open-ended NGS plasma testing for mcfDNA identified disseminated *M kansasii* infection much earlier than standard microbiology and thus helped in initiation and modification of pathogen directed treatment.

**Disclosures:**

**All Authors**: No reported disclosures

